# Association of obesity phenotypes with left ventricular mass index and left ventricular hypertrophy in children and adolescents

**DOI:** 10.3389/fendo.2022.1006588

**Published:** 2022-09-29

**Authors:** Simonetta Genovesi, Elena Tassistro, Marco Giussani, Giulia Lieti, Ilenia Patti, Antonina Orlando, Massimo Montemerlo, Laura Antolini, Gianfranco Parati

**Affiliations:** ^1^ School of Medicine and Surgery, University of Milano - Bicocca, Milan, Italy; ^2^ Cardiology Unit, Istituto Auxologico Italiano, Istituto di Ricovero e Cura a Carattere Scientifico (IRCCS), Milan, Italy

**Keywords:** adolescents, children, left ventricular mass index, left ventricular hypertrophy, metabolically healthy obesity, HOMA-index, uric acid

## Abstract

It has been argued that metabolically healthy obesity (MHO) does not increase the risk of cardiovascular disease. The aim of this study is to evaluate whether, in a population of obese children/adolescents, the metabolically unhealthy obesity (MUO) phenotype is associated with higher left ventricular mass index and/or higher prevalence of left ventricular hypertrophy than the MHO phenotype. We also tested whether the addition of an insulin resistance index (HOMA-index >90th percentile by sex and age) and the presence of hyperuricemia (serum uric acid >90th percentile by sex and age) to the definition of MUO better identified obese children with early cardiac damage. Left ventricular hypertrophy was defined as the presence of left ventricular mass index greater than or equal to the age- and sex-specific 95th percentile.

The study population included 459 obese children (males 53.2%, mean age 10.6 [standard deviation, 2.6] years), of whom 268 (58.4%) were MUO. The left ventricular mass index was higher in MUO children than in MHO children (37.8 vs 36.3 g/m^2.7^, p=0.015), whereas the percentage of MUO children presenting left ventricular hypertrophy was only slightly higher in MUO children (31.1 vs 40%, p=0.06). Multiple linear regression analyses showed that the variables significantly associated with higher left ventricular mass index were male gender (p<0.01), Body Mass Index z-score (p<0.001) and Waist-to-Height-ratio (p<0.001). Multiple logistic regression analyses showed that the presence of left ventricular hypertrophy was only significantly associated with higher Body Mass Index z-score (p<0.05) and Waist-to-Height-ratio (p<0.05). In spite of the higher left ventricular mass index of MUO as compared to MHO children, the MUO phenotype was not a significant predictor of either higher left ventricular mass index or higher left ventricular hypertrophy prevalence. The MUO phenotype had a low predictive ability on the presence of left ventricular hypertrophy. The area under the receiver operating characteristic curve was 0.57 (sensitivity 0.64, 1-specificity 0.55). The addition of insulin resistance and hyperuricemia to the definition of MUO did not change the results observed with the standard definition of MUO at multivariable analysis.

The MUO phenotype appears to be of little usefulness in identifying the early presence of cardiac damage in a large population of obese children and adolescents. Excess weight and abdominal obesity are confirmed as an important determinant of early organ damage in obese children.

## Introduction

Obesity in adults is often associated with several cardiovascular risk factors, such as those characterizing the metabolic syndrome: high blood pressure (BP), low HDL cholesterol, hypertriglyceridemia and hyperglycemia (1). Adults in severe excess weight who have none of these risk factors are called metabolically healthy obese (MHO). In contrast, the metabolically unhealthy obese (MUO) are those who have one or more of these risk factors (2, 3). Although there is some uncertainty in the definition of the different risk factors, these classifications are also used in pediatrics (4, 5). Damanhoury et al. (6) proposed that children with none of the following alterations are considered to have the MHO phenotype: systolic blood pressure (SBP) or diastolic blood pressure (DBP) ≥90th percentile, glycaemia ≥100mg/dl, HDL cholesterol ≤40mg/dl, triglycerides ≥100 mg/dl (children <10 years) or ≥130 mg/dl (children ≥10 years). A recent study showed that, in a population of obese children and adolescents, elevated serum uric acid (SUA) values and HOMA-index were able to predict the possibility of being MUO according to Damanhoury’s classification (6). In adults, the presence of cardiovascular risk factors is frequently associated with organ damage. In pediatric age, early organ damage has been described in at-risk individuals, and left ventricular hypertrophy (LVH) is the organ damage most frequently found in children and adolescents with cardiovascular risk factors (7, 8). Excess weight is an important determinant for the development of LVH in children and adolescents (9), but even hemodynamic and metabolic factors may contribute to the increase in cardiac mass (10–12). The aim of this study is to evaluate whether, in a population of obese children/adolescents, i) individuals defined as MUO according to the Damanhoury classification (6) have higher left ventricular mass index (LVMI) and/or higher prevalence of LVH than MHO individuals ii) the addition of higher-than-normal HOMA-index and SUA values to the four risk factors of the Damanhoury’s classification allows better identification of children with initial cardiac organ damage (i.e. higher LVMI and higher prevalence of LVH).

## Methods (for extended form see **supplementary material**)

### Subjects

We studied a cohort of 459 obese children and adolescents, consecutively referred from december 2012 to april 2022 by their primary care pediatricians to our clinic. Exclusion criteria were: impaired glucose tolerance, diabetes, any form of secondary hypertension, treatment with antihypertensive drugs.

### Anthropometric parameters and blood pressure measurements

In all children, height, weight and waist circumference (WC) were measured. Body mass index (BMI) and waist-to-height-ratio (WtHr) were calculated. BMI z-scores were calculated using the Centre for Disease and Control prevention charts. Obesity was defined according to the International Obesity Task Force (13). Children were classified as pre-pubertal and pubertal according to Tanner (14).

Blood pressure measurements were performed by an oscillometric device validated in children (Omron 705 IT; Omron Co, Kyoto, Japan). The BP measurement was performed 3 times and the average of the last two measurements was considered. Systolic BP (SBP) and diastolic BP (DBP) percentiles and z-scores were calculated according to the nomograms of the National High Blood Pressure Education Program (NHBPEP) Working Group on High Blood Pressure in Children and Adolescents (15).

### Biochemical parameters

Blood samples were taken from all subjects after a 12-hour fasting period in order to measure serum concentrations of high-density lipoprotein (HDL), triglycerides, glucose, insulin and uric acid. HOMA index was calculated by dividing the product of serum insulin (µU/ml) and serum glucose (mmol/L) by 22.5 (16).

### Echocardiography

Two-dimensional M-mode echocardiography images were obtained using digital echocardiography equipment (Aloka ProSound SSD Alpha 10, Tokyo, Japan) with 1-5MHz transducers, and following the recommendations for standard M-mode measurements. Left ventricular mass was calculated according to the American Society of Echocardiography convention, and indexed (LVMI) to height (m^2.7^) (17).

Left ventricular hypertrophy was defined as the presence of a LVMI greater than or equal to the 95^th^ percentile specific for age and gender, according to the reference values by Khoury (18) as recommended by the latest guidelines of the European Society of Cardiology (19).

### MHO/MUO definition

Standard MUO phenotype was defined as the presence of at least one of the following risk factors: SBP and/or DBP> 90^th^ percentile, glycaemia >100 mg/dl, HDL cholesterol <40 mg/dl, triglycerides >100 mg/dl (children <10 years) or >130 mg/dl (children>10 years) (6).

New MUO phenotype was defined as the presence of at least one of the following risk factors: SBP and/or DBP> 90^th^ percentile, glycaemia >100 mg/dl, HDL cholesterol <40 mg/dl, triglycerides >100 mg/dl (children <10 years) or >130 mg/dl (children>10 years). HOMA index value >90^th^ percentile by gender and age (20), and SUA value >90^th^ percentile by gender and age (21).

### Statistical analysis

Multiple linear regression models were used to assess the impact of standard (or new) classification, gender, age, pubertal status, BMI z-score (or WtHr) on LVMI.

Multiple logistic regression models were used to assess the impact of standard (or new) classification, gender, age, pubertal status, BMI z-score (or WtHr) on the presence of LVH.

To investigate the ability of the two classifications to discriminate among the presence/absence of LVH, sensitivity and 1-specificity were calculated. The probability of having LVH was related to the two classifications in a logistic regression model adjusted by gender, age, pubertal status and BMI to obtain a continuous score by the weighted contribution of the classification and the adjustment factors through the model coefficients. The receiver operating characteristic (ROC) curve of each score was calculated to investigate the ability of the classification to discriminate among the presence/absence of LVH. The area under the ROC curve (AUC) of each classification was calculated as summary discrimination measure.

Statistical analyses were performed with R 4.1.2 (http://www.R-project.org). All p-values were 2-sided, with p-values <0.05 considered statistically significant.

## Results

The study population was described in **Table 1**. We included 459 obese children (males 53.2%), of whom 40.7% had started pubertal development (**Table 1**). Forty-three percent had SBP and/or DBP values >90th percentile. Regarding metabolic disorders, only 1.3% had serum glucose >100 mg/dl, however 77.8% had HOMA index values >90th percentile. In 19.6% of the children, plasma triglyceride values were above the normal cut-off, and in 16.1% HDL cholesterol was <40 mg/dl. Serum uric acid levels were greater than the 90th percentile in 21.8% of the subjects. The mean LVMI value was 37.1 g/m^2.7^ and 36.3% of the population had LVH.

**Table 1:** Anthropometric and clinic characteristics of the study population.

		Standard classification			New classification		
Parameter	Overall (N = 459)	MHO (N = 191, 41.6%)	MUO (N = 268, 58.4%)	P	MHO (N = 45, 9.8%)	MUO (N = 414, 90.2%)	P
Age (years), mean (SD)	10.6 (2.6)	9.8 (2.3)	11.1 (2.7)	<0.001	9.7 (2.6)	10.6 (2.6)	0.018
Gender (males), n (%)	244 (53.2)	89 (46.6)	155 (57.8)	0.022	21 (46.7)	223 (53.9)	0.446
Puberty yes, n (%)	185 (40.7)	63 (33.2)	122 (46.0)	0.008	13 (28.9)	172 (42.0)	0.125
Weight (kg), median (Q1-Q3)	56.9 (44.7, 72.9)	52.5 (41.1, 62.5)	63.7 (48.8, 79.1)	<0.001	45.9 (38.5-61.5)	59.2 (45.4-73.7)	<0.001
Height (cm), mean (SD)	145.9 (15.1)	141.8 (13.8)	148.0 (15.2)	<0.001	140.2 (15.5)	146.5 (14.9)	0.007
BMI, mean (SD)	27.5 (4.1)	25.9 (3.0)	28.6 (4.4)	<0.001	24.8 (3.3)	27.8 (4.1)	<0.001
BMI (z-score), median (Q1-Q3)	2.1 (1.9, 2.3)	2.0 (1.9, 2.2)	2.2 (2.0, 2.4)	<0.001	2.0 (1.9, 2.1)	2.1 (2.0, 2.3)	<0.001
Waist (cm), median (Q1-Q3)	82.5 (76.0-91.0)	79.5 (73.4, 85.0)	87.0 (79.4, 94.0)	<0.001	78.0 (10.0)	84.9 (11.0)	<0.001
WtHr (%), median (SD)	57.3 (54.3-60.9)	56.0 (53.5-59.0)	58.2 (54.7-61.4)	<0.001	55.0 (53.0, 58.0)	57.5 (54.4, 61.1)	0.001
WtHr > 50%, n (%)	443 (96.5)	185 (96.9)	258 (96.3)	0.935	44 (97.8)	399 (96.4)	0.953
Systolic BP (mmHg), mean (SD)	115.9 (13.1)	107.3 (7.8)	122.1 (12.6)	<0.001	105.8 (7.3)	117.0 (13.1)	<0.001
Diastolic BP (mmHg), mean (SD)	68.4 (8.6)	64.2 (6.1)	71.4 (8.9)	<0.001	64.1 (5.6)	68.9 (8.7)	<0.001
Systolic BP (z-score), mean (SD)	1.1 (1.0)	0.4 (0.6)	1.5 (1.0)	<0.001	0.3 (0.6)	1.1 (1.0)	<0.001
Diastolic BP (z-score), mean (SD)	0.6 (0.7)	0.3 (0.5)	0.8 (0.7)	<0.001	0.3 (0.5)	0.6 (0.7)	0.003
SBP and/or DBP > 90^th^ percentile, n (%)	141 (30.7)	0 (0.0)	141 (52.6)	–	0 (0.0)	141 (34.0)	–
Glucose (mg/dl), mean (SD)	84.1 (7.2)	83.6 (7.2)	84.4 (7.2)	0.220	79.9 (6.0)	84.5 (7.2)	<0.001
Glucose ≥ 100 mg/dl, n (%)	6 (1.3)	0 (0.0)	6 (2.2)	–	0 (0.0)	6 (1.4)	–
Triglycerides (mg/dl), median (Q1-Q3)	75.0 (55.0, 104.0)	64.0 (50.5, 81.5)	85.5 (61.0, 135.3)	<0.001	50.0 (40.0, 65.0)	77.0 (57.3, 110.0)	<0.001
Triglycerides ≥ 100 mg/dl or ≥ 130 mg/dl*, n (%)	90 (19.6)	0 (0.0)	90 (33.6)	–	0 (0.0)	90 (21.7)	–
HDL cholesterol (mg/dl), median (Q1-Q3)	49.0 (42.0, 56.0)	52.0 (47.0, 58.0)	45.5 (38.0, 53.0)	<0.001	55.0 (48.0, 63.0)	48.0 (41.0, 54.0)	<0.001
HDL cholesterol < 40 mg/dl, n (%)	74 (16.1)	0 (0.0)	74 (27.6)	–	0 (0.0)	74 (17.9)	–
Uric acid (mg/dl), median (Q1-Q3)	4.5 (3.8, 5.3)	4.3 (3.6, 4.9)	4.7 (4.1, 5.5)	<0.001	4.0 (3.3, 4.5)	4.6 (3.9, 5.4)	<0.001
Uric acid (mg/dl) > 90^th^ percentile, n (%)	100 (21.8)	31 (16.2)	69 (25.7)	0.020	0 (0.0)	100 (24.2)	–
HOMA Index^$^, median (Q1-Q3)	3.0 (2.1, 4.5)	2.6 (1.9, 3.8)	3.2 (2.3, 4.9)	<0.001	1.5 (1.1, 1.8)	3.1 (2.3, 4.7)	<0.001
HOMA Index > 90^th^ percentile, n (%)	357 (77.8)	139 (72.8)	218 (81.3)	0.039	0 (0.0)	357 (86.2)	–
LVMI (g/m^2.7^), median (Q1-Q3)	37.1 (32.7, 41.8)	36.3 (32.4, 40.4)	37.8 (33.3, 42.4)	0.015	35.8 (32.5, 42.1)	37.3 (32.9, 41.7)	0.610
LVH, n (%)	165 (36.3)	59 (31.1)	106 (40.0)	0.063	16 (35.6)	149 (36.3)	0.999

SD, standard deviation; Q1, first quartile; Q3, third quartile. BMI, body mass index; WtHr, waist-to-height-ratio; LVMI, left ventricular mass index; LVH, left ventricular hypertrophy.

*Triglycerides ≥ 100 if children < 10 years or ≥ 130 if children ≥ 10 years.

^$^Calculated as plasma insulin (mU/ml) * plasma glucose (mmol/l)/22.5.

Considering the standard definition of the MUO phenotype, 268 (58.4%) children were found to be MUO, whereas the percentage increased significantly using the new classification (n = 414, 90.2%). Specifically, 72.8% of the children classified as MHO with the standard classification had HOMA-index values >90th percentile and 16.2% had SUA values >90th percentile.

**Figure 1:**
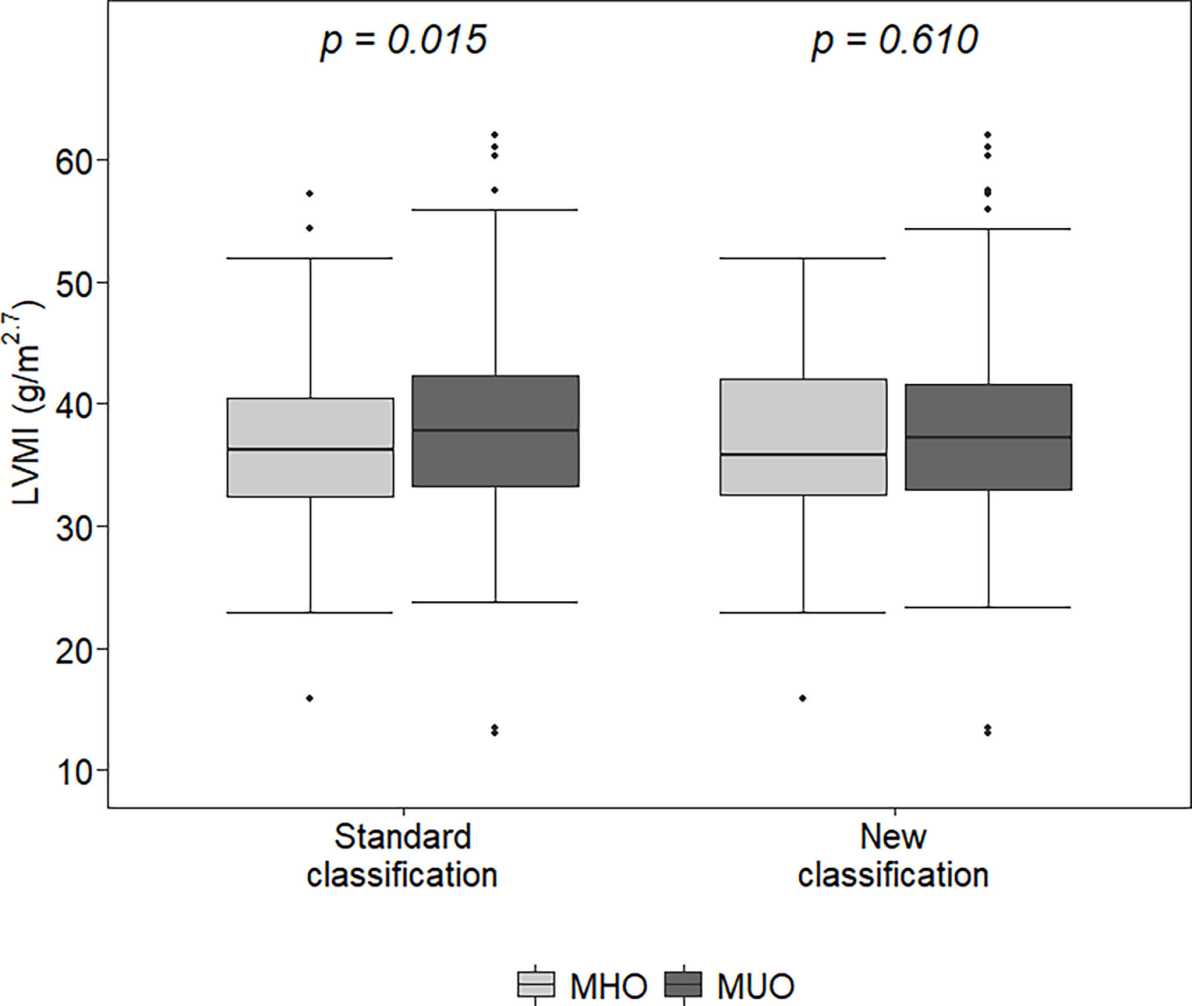
Left ventricular mass index (LVMI) in metabolically healthy obese (MHO) and metabolically unhealthy obese (MUO) according to the two classifications. The lower (and upper) whiskers extend up to the lowest (highest) datum still within 1.5 times IQR of the first (third) quartile.

With both classifications, MUO children were older (p<0.02) and had higher BMI z-scores and WtHr values than MHO children (p<0.001). The left ventricular mass index was higher in MUO children than in MHO children (37.8 vs 36.3 g/m^2.7^, p=0.015) when defined by the standard classification, but there were no differences between the LVMI values of MHO and MUO children when defined by the new classification (35.8 vs 37.3 g/m^2.7^, p=0.61) (**Figure 1**). Similar results were observed regarding the percentage of children with LVH (31.1 vs 40%, p=0.06 MHO vs MUO, standard classification and 35.6 vs 36.3% p=0.99 MHO vs MUO, new classification) (**Table 1**).

Multiple linear regression analyses showed that the variables significantly associated with higher LVMI were male gender (p<0.01), BMI z-score (p<0.001, **Table 2**, Model A) and WtHr (p<0.001, **Table 2**, Model B), both when the models were adjusted for standard and new MUO classification. Adjusting for WtHr (Model B), an inverse association with age was present with both classifications (p<0.01). Results of the same models after the log-transformation of the outcome were similar(data not shown).

**Table 2:** Effect of metabolically unhealthy obese phenotype, gender, age, puberty, BMI (Model A) or waist-to-height ratio (Model B) on left ventricular mass index by a multiple linear regression model.

Standard classification							
Variable	Model A - BMI				Model B – Waist-to-Height Ratio		
	b	(95% CI)	P		b	(95% CI)	P
Intercept	26.965	(20.512; 33.418)	<0.001		24.399	(16.849; 31.948)	<0.001
MUO vs MHO	1.035	(-0.264; 2.335)	0.118		1.151	(-0.139; 2.442)	0.080
Gender (males)	2.054	(0.742; 3.367)	0.002		2.306	(1.010; 3.601)	0.001
Age (years)	-0.232	(-0.581; 0.116)	0.191		-0.527	(-0.869; -0.184)	0.003
Puberty	0.590	(-1.157; 2.338)	0.507		1.458	(-0.335; 3.251)	0.111
BMI (z-score)	5.122	(2.756; 7.487)	<0.001		–	–	–
WtHr	–	–	–		0.280	(0.150; 0.410)	<0.001
New classification							
Variable	Model A - BMI				Model B – Waist-to-Height Ratio		
	b	(95% CI)	P		b	(95% CI)	P
Intercept	26.162	(19.761; 32.562)	<0.001		23.442	(15.908; 30.975)	<0.001
MUO vs MHO	-0.139	(-2.231; 1.953)	0.896		0.192	(-1.879; 2.262)	0.856
Gender (males)	2.093	(0.777; 3.408)	0.002		2.373	(1.075; 3.671)	<0.001
Age (years)	-0.169	(-0.512; 0.174)	0.333		-0.482	(-0.821; -0.142)	0.006
Puberty	0.569	(-1.184; 2.321)	0.524		1.479	(-0.322; 3.280)	0.107
BMI (z-score)	5.518	(3.141; 7.895)	<0.001		–	–	–
WtHr	–	–	–		0.296	(0.166; 0.426)	<0.001

b, indicates multivariate coefficient; CI, confidence interval; MUO, metabolically unhealthy obese; MHO, metabolically healthy obese; BMI, body mass index; WtHr, waist-to-height ratio.

Multiple logistic regression analyses showed that the presence of LVH was only significantly associated with BMI z-score (p<0.05, **Table 3**, Model A) and WtHr (p<0.05, **Table 3**, Model B), both when the models were adjusted for the standard and the new classification.

**Table 3:** Effect of metabolically unhealthy obese phenotype, gender, age, puberty, BMI (Model A) or waist-to-height ratio (Model B) on left ventricular hypertrophy by a multiple logistic regression model.

Standard classification							
Variable	Model A - BMI				Model B – Waist-to-Height Ratio		
	OR	(95% CI)	P		OR	(95% CI)	P
MUO vs MHO	1.415	(0.933; 2.157)	0.104		1.417	(0.936; 2.155)	0.101
Gender (males)	0.914	(0.601; 1.390)	0.674		0.940	(0.621; 1.422)	0.769
Age (years)	0.971	(0.869; 1.084)	0.598		0.924	(0.828; 1.030)	0.155
Puberty	1.163	(0.664; 2.037)	0.595		1.371	(0.774; 2.437)	0.280
BMI (z-score)	2.146	(1.020; 4.633)	0.047		–	–	–
WtHr	–	–	–		1.054	(1.012; 1.099)	0.012
New classification							
Variable	Model A - BMI				Model B – Waist-to-Height Ratio		
	OR	(95% CI)	P		OR	(95% CI)	P
MUO vs MHO	0.884	(0.459; 1.751)	0.716		0.915	(0.478; 1.802)	0.792
Gender (males)	0.925	(0.609; 1.404)	0.716		0.959	(0.635; 1.448)	0.841
Age (years)	0.992	(0.890; 1.106)	0.892		0.938	(0.841; 1.044)	0.243
Puberty	1.157	(0.662; 2.022)	0.607		1.385	(0.783; 2.458)	0.263
BMI (z-score)	2.495	(1.176; 5.455)	0.019		–	–	–
WtHr	–	–	–		1.060	(1.018; 1.105)	0.005

OR, indicates odds ratio; CI, confidence interval; MUO, metabolically unhealthy obese; MHO, metabolically healthy obese; BMI, body mass index; WtHr, waist-to-height ratio.

Additional analyses performed separately in pre-pubertal and pubertal individuals and in males and females confirmed these findings (**Tables S1–S4**).

**Figure 2:**
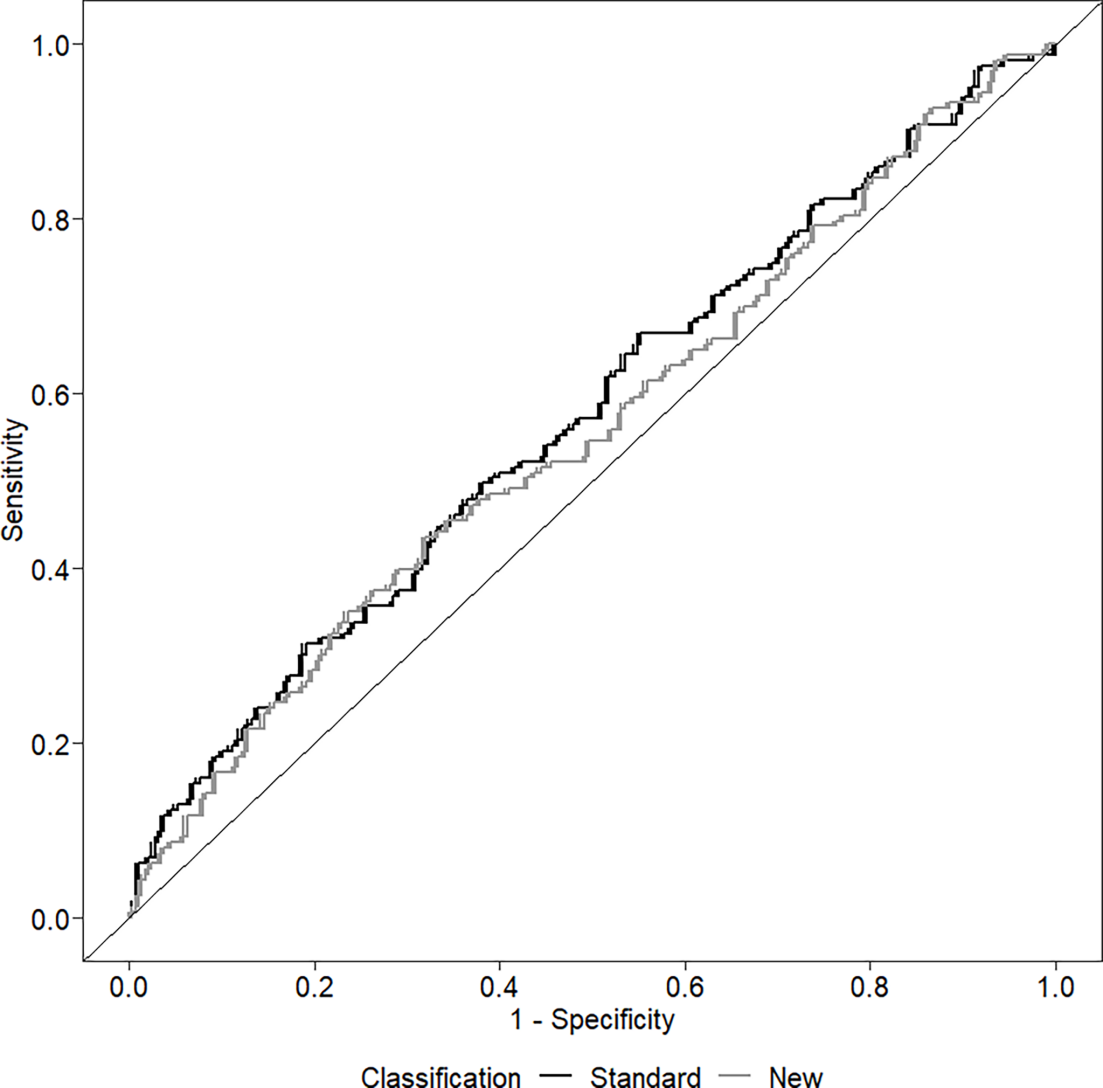
Receiver operating curve (ROC) investigating the ability of the two classifications to discriminate among the presence/absence of left ventricular hypertrophy.

The ability of the two classifications to predict the presence of LVH is shown in **Figure 2**. The AUC of the standard classification was 0.574, while that of the new classification was 0.557. Considering sensitivity, among the 165 children with LVH, 106 (64.2%) were classified as MUO with the standard classification, and 149 (90.3%) with the new classification. In contrast, among the 290 children without LVH, 159 (54.8%) were classified as MUO with the standard classification and 261 (90.0%) with the new classification. **Table 4** shows sensitivity, specificity, positive and negative predictive values of the two classifications.

**Table 4:** Predictive capability of the two classifications to discriminate among the presence/absence of left ventricular hypertrophy.

Standard classification		
	Specificity (45.2%)	Sensitivity (64.2%)
Negative predictive value (68.6%)	131 TN	59 FN
Positive predictive value (39.6%)	159 FP	106 TP
New classification		
	Specificity (10.0%)	Sensitivity (90.3)
Negative predictive value (64.4%)	29 TN	16 FN
Positive predictive value (36.0%)	261 FP	149 TP

FN, false negative; FP, false positive; TN, true negative; TP, true positive.

## Discussion

The prevalence of the MUO phenotype in our population of obese children is high. About 60% of the sample is found to have at least one of the criteria included in the standard classification and about 90% have the MUO phenotype considering the new classification. However, both classifications show poor predictive value in identifying children and adolescents with higher LVMI or LVH.

### MUO phenotype and cardiac damage: standard classification

In adults, the prevalence of unhealthy obesity ranges from approximately 66 to 91% (22). Regarding pediatric age, the prevalence of the MUO phenotype reported in the literature varies widely (32 to 80%) (23). In our population, when using the standard classification, we find a prevalence of MUO children that is in agreement with that described by Reinehr et al. in a large sample of Caucasian children (24), while it is lower than that reported by other authors in a smaller sample (4).

The MUO children in our sample are older than the MHO children, more often have started pubertal development and more often are male. Both BMI z-score and WtHr are higher in the MUO group. The prevalence of elevated BP in the MUO phenotype is 53%, approximately 30% have dyslipidaemia and only 2% have elevated plasma glucose values. Left ventricular mass index is significantly higher in MUO subjects than in MHO subjects and there is a slightly higher prevalence of LVH.

In multivariable analysis models, the MUO phenotype is associated neither with higher LVMI nor with higher prevalence of LVH, whereas BMI z-score and WtHr are associated with both. The risk of presenting LVH doubles for each BMI z-score point and increases by 5% for each WtHr point.

### MUO phenotype and cardiac damage: New classification

If we add an insulin resistance index and the presence of hyperuricemia to the standard definition of MUO, the prevalence of the phenotype almost doubles. This fact leads us to question whether we can really define ‘healthy’ children and adolescents according to Damanhoury’s classification (6).This classification significantly underestimates children with early IR. In the group defined as MHO, compared with 1.6% high plasma glucose levels, 73% have HOMA-index values above the 90^th^ percentile. In addition, 16% had elevated SUA values. These data confirm what has already been observed in another population (25) and suggest the presence of a high percentage of metabolic alterations in obese children and adolescents that is not recognised by the standard definition of MUO.

The MUO phenotype is more frequent in older individuals and those with greater excess weight and waist circumference. With the new classification, no significant differences in LVMI and LVH rates are observed between the two phenotypes, but this could also be due to the fact that the MHO group is poorly represented (less than 10%).

In the multivariable model, the main determinants of cardiac damage (i.e. elevated LVMI and presence of LVH) are BMI and WtHr values: the risk of LVH is two and a half times higher for a one-point increase in the BMI z-score and increases by 6% for each point of WtHr.

### Predictive value of the two classifications on LVH

Both classifications have a modest ability to predict the presence of LVH. The area under the ROC curve has a low value for both classifications. The new classification has a high sensitivity, but at the price of very poor specificity, while the standard classification has both poor sensitivity and poor specificity. It therefore seems that, regarding the identification of early cardiac damage in obese children, there is no advantage in considering a cluster of risk factors, rather than assessing the impact of excess weight (or visceral adiposity) as a single risk factor. This result could depend on several causes. Firstly, the impact of different risk factors on early organ damage could differ depending on the organ considered. An increased carotid intima media thickness (cIMT), associated with the MUO phenotype, has been described in children (26). It is possible that metabolic alterations exert a negative effect more on the vessels than on the heart and that this is mediated by the presence of endothelial dysfunction (27, 28). It would be interesting to assess whether adding IR and hyperuricemia to the definition of the MUO phenotype could improve the identification of children with early vascular organ damage. In addition, it is known that BMI is the main determinant of the onset of LVH in children and has an effect three times greater than that of hypertension (29), while the effect of high BP values on vascular stiffness is much more important than that of excess weight (30).

Secondly, it is possible that if the study population included children with a lower degree of excess weight, the MUO phenotype (however defined) might be more useful in identifying early organ damage. Indeed, in this case, we might expect fewer subjects identified as MUO and this might help to better discriminate those at greater risk of developing organ damage.

Third, as in adults the MUO phenotype has been associated with a higher prevalence of LVH than MHO (31, 32),it is possible that the negative impact of BP and metabolic alterations on the heart needs time to manifest itself and does not do so as early as childhood.

### Conclusions

The standard classification of the MUO phenotype appears to be of little use in identifying the early presence of cardiac damage in a large population of obese children and adolescents. In addition, most obese individuals classified as MHO have significant metabolic alterations that are worthy of the paediatric physician’s attention. Excess weight and abdominal obesity are confirmed as an important determinant of early cardiac damage in obese children

## Data availability statement

The raw data supporting the conclusions of this article will be made available by the authors, without undue reservation.

## Ethics statement

The studies involving human participants were reviewed and approved by Ethic Committee Name: Istituto Auxologico Italiano, Approval Code: 2015102002, Approval Date: 20/11/2015. Written informed consent to participate in this study was provided by the participants’ legal guardian/next of kin.

## Author contributions

SG conceptualized and designed the study, drafted the initial manuscript, and reviewed and revised the manuscript. IP, GL, AO, MG and MM collected data. ET and LA performed data analysis. GP reviewed and revised the manuscript. All authors approved the final manuscript as submitted and agree to be accountable for all aspects of the work.

## Funding

This research was funded by the Italian Ministry of Health.

## Conflict of interest

The authors declare that the research was conducted in the absence of any commercial or financial relationships that could be construed as a potential conflict of interest.

## Publisher’s note

All claims expressed in this article are solely those of the authors and do not necessarily represent those of their affiliated organizations, or those of the publisher, the editors and the reviewers. Any product that may be evaluated in this article, or claim that may be made by its manufacturer, is not guaranteed or endorsed by the publisher.
