# Supplementary material for: Association of obesity phenotypes with left ventricular mass index and left ventricular hypertrophy in children and adolescents
Source: Front Endocrinol (Lausanne). 2022 Sep 29;13:1006588. doi: 10.3389/fendo.2022.1006588 (PMC9558292; doi:10.3389/fendo.2022.1006588)
Supplement: Supplementary file 1 [file DataSheet_1.docx]

# Methods

## Subjects

We studied a cohort of 459 obese children and adolescents (mean age 10.6 years [standard deviation, SD 2.6]), consecutively referred from december 2012 to april 2022 by their primary care pediatricians to our Unit for Cardiovascular Risk Assessment in Children. Exclusion criteria were: impaired glucose tolerance, diabetes, any form of secondary hypertension, treatment with antihypertensive drugs.

## Anthropometric parameters and blood pressure measurements

In all children, height, weight and waist circumference (WC) were measured. Weight was approximated to the nearest 100 grams, and height to the nearest 1 mm. Waist circumference was measured in standing position to the nearest 0.5 cm by a non-elastic flexible tape positioned midway between the last rib and the iliac crest. Body mass index (BMI) was calculated as weight (kg)/height (m)^2^. Waist-to-height-ratio (WtHr) was calculated dividing WC by height. BMI z-scores were calculated using the Centre for Disease and Control prevention charts available at https://www.cdc.gov/growthcharts/clinical_charts.htm. The presence of obesity was defined according to the International Obesity Task Force classification (13). Pubertal stage was assessed by a medical examination and children were classified into two categories: pre-pubertal and pubertal according to Tanner (14), considering pre-pubertal boys with gonadal stage 1 and girls with breast stage 1.

Blood pressure measurements were performed by an oscillometric device validated in children (Omron 705 IT; Omron Co, Kyoto, Japan), with the appropriate cuff for the children’s upper-arm size, after at least 5 minutes of rest, with the child in a sitting position. The BP measurement was performed 3 times (at intervals of a few minutes) and the average of the last two measurements was considered. Systolic BP (SBP) and diastolic BP (DBP) percentiles and z-scores were calculated according to the nomograms of the National High Blood Pressure Education Program (NHBPEP) Working Group on High Blood Pressure in Children and Adolescents (15).

## Biochemical parameters

Blood samples were taken from all subjects after a 12-hour fasting period in order to measure serum concentrations of high-density lipoprotein (HDL), triglycerides, glucose, insulin, uric acid. Commercial kits, normally used for routine examinations of patients, were employed for all analyses. In detail: colorimetric enzymatic test in homogeneous phase HDL-Cholesterol Gen.4 Cobas Roche for HDL cholesterol; enzymatic colorimetric test Triglycerides Cobas Roche for triglycerides assay; enzymatic method with hexokinase Glucose HK Gen.3 Cobas Roche, for glucose assay; immunoAssay in ElectroChemiLuminescence Elecsys Insulin Cobas Roche for insulin assay; colorimetric enzymatic test Uric Acid 2 Cobas Roche for uric acid assay. HOMA index was calculated by dividing the product of serum insulin (µU/ml) and serum glucose (mmol/L) by 22.5 (16).

## Echocardiography

Two-dimensional M-mode echocardiography images were obtained in the standard precordial positions using digital echocardiography equipment (Aloka ProSound SSD Alpha 10, Tokyo, Japan) with 1-5MHz transducers, and following the recommendations for standard M-mode measurements. Instantaneous measurements were made over three cardiac cycles and the average values were obtained from each subject for: left atrial end-diastolic diameter (LAd), interventricular septum thickness at end-diastole (IVSd), left ventricular posterior wall thickness at end-diastole (LVPWd) and left ventricular end-diastolic diameter (LVEDd). Left ventricular mass was calculated according to the American Society of Echocardiography convention, and indexed (LVMI) to height (m2.7) (17).

Left ventricular hypertrophy was defined as the presence of a LVMI greater than or equal to the 95th percentile specific for age and gender, according to the reference values by Khoury (18) as recommended by the latest guidelines of the European Society of Cardiology (19).

## MHO/MUO definition

We considered as standard definition of MHO phenotype the following: SBP and DBP <90^th^ percentile by gender, age and height percentile, glycemia <100 mg/dl, HDL cholesterol *>*40 mg/dl, triglycerides<100 mg/dl (children <10 years) or<130 mg/dl (children>10 years) (6).

Standard MUO phenotype was defined as the presence of at least one of the following risk factors: SBP and/or DBP> 90th percentile, glycaemia >100 mg/dl, HDL cholesterol *<*40 mg/dl, triglycerides >100 mg/dl (children <10 years) or >130 mg/dl (children>10 years) (6).

We considered as new definition of MHO phenotype the following: SBP and DBP <90^th^ percentile by gender, age and height percentile, glycaemia <100 mg/dl, HDL cholesterol *>*40 mg/dl, triglycerides<100 mg/dl (children <10 years) or<130 mg/dl (children>10 years), HOMA index value <90^th^ percentile by gender and age (20), and SUA value <90^th^ percentile by gender and age (21).

New MUO phenotype was defined as the presence of at least one of the following risk factors: SBP and/or DBP> 90th percentile, glycemia >100 mg/dl, HDL cholesterol *<*40 mg/dl, triglycerides >100 mg/dl (children <10 years) or >130 mg/dl (children>10 years). HOMA index value >90^th^ percentile by gender and age (20), and SUA value >90^th^ percentile by gender and age (21).

## Statistical analysis

The characteristics of the cohort, overall and stratified according to the standard and the new classification, were described by mean and standard deviation (SD) or median and first-third quartiles (Q1-Q3) if the variables were continuous, by frequencies and percentages if they were categorical. Univariate analyses to compare the characteristics of the MHO and MUO children were conducted through the t-test or the Mann-Whitney test in case of continuous variables, and through the Chi-Square test in case of categorical variables.

The distribution of LVMI in MHO and MUO children identified with both the standard and the new classification was represented by boxplots and the groups were compared through the Mann-Whitney test.

Multiple linear regression models were used to assess the impact of standard (or new) classification, gender, age, pubertal status, BMI z-score (or WtHr) on LVMI.

Multiple logistic regression models were used to assess the impact of standard (or new) classification, gender, age, pubertal status, BMI z-score (or WtHr) on the presence of LVH.

To investigate the ability of the two classifications to discriminate among the presence/absence of LVH, sensitivity and 1-specificity were calculated. Sensitivity was obtained by the proportion of individuals classified as MUO among those with LVH. The complement to one of specificity was obtained by the proportion of individuals classified as MUO among those without LVH. The probability of having LVH was related to the two classifications in a logistic regression model adjusted by gender, age, pubertal status and BMI. This enabled us to obtain a continuous score by the weighted contribution of the classification and the adjustment factors through the model coefficients. The receiver operating characteristic (ROC) curve of each score was calculated to investigate the ability of the classification (and the adjustment factors) to discriminate among the presence/absence of LVH. The area under the ROC curve (AUC) of each classification was calculated as summary discrimination measure.

Statistical analyses were performed with R 4.1.2 ( <http://www.R-project.org> ). All p-values were 2-sided, with p-values <0.05 considered statistically significant.

**Table S1**. Effect of metabolically unhealthy obese phenotype, gender, age, BMI (Model A) or waist-to-height ratio (Model B) on left ventricular mass index separately in children that started pubertal development (left panel) and that not yet started it (right panel) by a multiple linear regression model.

| **Pubertal status (N = 185)** | | | | | | | | **Pre-pubertal status (N = 270)** | | | | | | |
| --- | --- | --- | --- | --- | --- | --- | --- | --- | --- | --- | --- | --- | --- | --- |
| **Standard classification** | | | | | | | | | | | | | | |
| **Variable** | **Model A- BMI** | | |  | **Model B - WtHr** | | | **Model A- BMI** | | |  | **Model B - WtHr** | | |
|  | **b** | **(95% CI)** | **P** |  | **b** | **(95% CI)** | **P** | **b** | **(95% CI)** | **P** |  | **b** | **(95% CI)** | **P** |
| Intercept | 27.435 | (16.921; 37.949) | <0.001 |  | 24.211 | (12.852; 35.571) | <0.001 | 27.769 | (18.801; 36.737) | <0.001 |  | 25.101 | (14.841; 35.362) | <0.001 |
| MUO vs MHO | 1.175 | (-0.964; 3.313) | 0.280 |  | 1.320 | (-0.751; 3.390) | 0.210 | 0.967 | (-0.702; 2.635) | 0.255 |  | 0.999 | (-0.666; 2.663) | 0.239 |
| Gender (males) | 1.201 | (-0.736; 3.138) | 0.223 |  | 1.257 | (-0.651; 3.166) | 0.195 | 2.772 | (0.941; 4.604) | 0.003 |  | 3.264 | (1.483; 5.044) | <0.001 |
| Age (years) | -0.160 | (-0.666; 0.345) | 0.533 |  | -0.253 | (-.0763; 0.256) | 0.328 | -0.331 | (-0.835; 0.173) | 0.197 |  | -0.774 | (-1.242; -0.306) | 0.001 |
| BMI (z-score) | 4.878 | (0.403; 9.353) | 0.033 |  | - | - | - | 4.990 | (2.005; 7.974) | 0.001 |  | - | - | - |
| WtHr | - | - | - |  | 0.254 | (0.060; 0.449) | 0.011 | - | - | - |  | 0.298 | (0.123; 0.473) | 0.001 |
| **New classification** | | | | | | | | | | | | | | |
| **Variable** | **Model A- BMI** | | |  | **Model B - WtHr** | | | **Model A- BMI** | | |  | **Model B - WtHr** | | |
|  | **b** | **(95% CI)** | **P** |  | **b** | **(95% CI)** | **P** | **b** | **(95% CI)** | **P** |  | **b** | **(95% CI)** | **P** |
| Intercept | 25.583 | (15.226; 35.940) | <0.001 |  | 22.401 | (11.009; 33.793) | <0.001 | 27.273 | (18.315; 36.231) | <0.001 |  | 24.580 | (14.326; 34.835) | <0.001 |
| MUO vs MHO | 0.405 | (-3.238; 4.048) | 0.827 |  | 0.505 | (-3.108; 4.118) | 0.783 | -0.328 | (-2.948; 2.291) | 0.805 |  | 0.113 | (-2.450; 2.676) | 0.931 |
| Gender (males) | 1.219 | (-0.724; 3.162) | 0.217 |  | 1.314 | (-0.600; 3.229) | 0.177 | 2.815 | (0.980; 4.649) | 0.003 |  | 3.339 | (1.559; 5.120) | <0.001 |
| Age (years) | -0.096 | (-0.590; 0.397) | 0.700 |  | -0.186 | (-0.686; 0.315) | 0.465 | -0.270 | (-0.776; 0.237) | 0.296 |  | -0.744 | (-1.211; -0.277) | 0.002 |
| BMI (z-score) | 5.570 | (1.259; 9.880) | 0.012 |  | - | - | - | 5.312 | (2.238; 8.386) | 0.001 |  | - | - | - |
| WtHr | - | - | - |  | 0.278 | (0.086; 0.470) | 0.005 | - | - | - |  | 0.309 | (0.132; 0.486) | 0.001 |

b indicates multivariate coefficient; CI, confidence interval; MUO, metabolically unhealthy obese; MHO, metabolically healthy obese; BMI, body mass index; WtHr, waist-to-height ratio

**Table S2**. Effect of metabolically unhealthy obese phenotype, gender, age, BMI (Model A) or waist-to-height ratio (Model B) on left ventricular hypertrophy separately in children that started pubertal development (left panel) and that not yet started it (right panel) by a multiple logistic regression model.

| **Pubertal status (N = 185)** | | | | | | | | **Pre-pubertal status (N = 270)** | | | | | | |
| --- | --- | --- | --- | --- | --- | --- | --- | --- | --- | --- | --- | --- | --- | --- |
| **Standard classification** | | | | | | | | | | | | | | |
| **Variable** | **Model A- BMI** | | |  | **Model B - WtHr** | | | **Model A- BMI** | | |  | **Model B - WtHr** | | |
|  | **OR** | **(95% CI)** | **P** |  | **OR** | **(95% CI)** | **P** | **OR** | **(95% CI)** | **P** |  | **OR** | **(95% CI)** | **P** |
| MUO vs MHO | 1.264 | (0.626; 2.593) | 0.516 |  | 1.246 | (0.627; 2.516) | 0.534 | 1.522 | (0.905; 2.577) | 0.115 |  | 1.526 | (0.907; 2.583) | 0.113 |
| Gender (males) | 0.763 | (0.399; 1.442) | 0.408 |  | 0.754 | (0.396; 1.417) | 0.383 | 1.034 | (0.584; 1.834) | 0.910 |  | 1.122 | (0.643; 1.967) | 0.686 |
| Age (years) | 0.966 | (0.817; 1.139) | 0.680 |  | 0.947 | (0.799; 1.120) | 0.527 | 0.976 | (0.834; 1.141) | 0.760 |  | 0.900 | (0.777; 1.041) | 0.158 |
| BMI (z-score) | 1.913 | (0.443; 8.280) | 0.382 |  | - | - | - | 2.378 | (0.948; 6.280) | 0.071 |  | - | - | - |
| WtHr | - | - | - |  | 1.049 | (0.984; 1.120) | 0.144 | - | - | - |  | 1.060 | (1.004; 1.120) | 0.035 |
| **New classification** | | | | | | | | | | | | | | |
| **Variable** | **Model A- BMI** | | |  | **Model B - WtHr** | | | **Model A- BMI** | | |  | **Model B - WtHr** | | |
|  | **OR** | **(95% CI)** | **P** |  | **OR** | **(95% CI)** | **P** | **OR** | **(95% CI)** | **P** |  | **OR** | **(95% CI)** | **P** |
| MUO vs MHO | 1.212 | (0.371; 4.676) | 0.760 |  | 1.198 | (0.367; 4.618) | 0.774 | 0.745 | (0.336; 1.702) | 0.473 |  | 0.807 | (0.371; 1.816) | 0.594 |
| Gender (males) | 0.766 | (0.401; 1.447) | 0.414 |  | 0.760 | (0.400; 1.427) | 0.396 | 1.046 | (0.593; 1.850) | 0.878 |  | 1.157 | (0.666; 2.022) | 0.607 |
| Age (years) | 0.978 | (0.831; 1.149) | 0.784 |  | 0.958 | (0.810; 1.129) | 0.606 | 1.009 | (0.862; 1.180) | 0.911 |  | 0.916 | (0.791; 1.058) | 0.235 |
| BMI (z-score) | 2.152 | (0.529; 8.815) | 0.282 |  | - | - | - | 2.897 | (1.107; 8.068) | 0.035 |  | - | - | - |
| WtHr | - | - | - |  | 1.053 | (0.989; 1.123) | 0.110 | - | - | - |  | 1.067 | (1.011; 1.129) | 0.020 |

OR indicates odds ratio; CI, confidence interval; MUO, metabolically unhealthy obese; MHO, metabolically healthy obese; BMI, body mass index; WtHr, waist-to-height ratio

**Table S3**. Effect of metabolically unhealthy obese phenotype, puberty, age, BMI (Model A) or waist-to-height ratio (Model B) on left ventricular mass index separately in males (left panel) and females (right panel) by a multiple linear regression model.

| **Males (N = 244)** | | | | | | | | **Females (N = 215)** | | | | | | |
| --- | --- | --- | --- | --- | --- | --- | --- | --- | --- | --- | --- | --- | --- | --- |
| **Standard classification** | | | | | | | | | | | | | | |
| **Variable** | **Model A- BMI** | | |  | **Model B - WtHr** | | | **Model A- BMI** | | |  | **Model B - WtHr** | | |
|  | **b** | **(95% CI)** | **P** |  | **b** | **(95% CI)** | **P** | **b** | **(95% CI)** | **P** |  | **b** | **(95% CI)** | **P** |
| Intercept | 29.779 | (20.996; 38.563) | <0.001 |  | 30.467 | (20.186; 40.748) | <0.001 | 25.866 | (15.632; 36.100) | <0.001 |  | 20.008 | (8.438; 31.577) | 0.001 |
| MUO vs MHO | 0.520 | (-1.262; 2.302) | 0.566 |  | 0.806 | (-0.971; 2.583) | 0.372 | 1.518 | (-0.409; 3.444) | 0.122 |  | 1.403 | (-0.502; 3.307) | 0.148 |
| Puberty | -0.258 | (-2.514; 1.999) | 0.822 |  | 0.460 | (-1.872; 2.791) | 0.698 | 1.646 | (-1.133; 4.426) | 0.244 |  | 2.682 | (-0.142; 5.507) | 0.063 |
| Age (years) | -0.172 | (-0.639; 0.295) | 0.469 |  | -0.463 | (-0.928; 0.001) | 0.050 | -0.323 | (-0.857; 0.211) | 0.235 |  | -0.619 | (-1.138; -0.100) | 0.020 |
| BMI (z-score) | 4.758 | (1.774; 7.743) | 0.002 |  | - | - | - | 5.723 | (1.849; 9.597) | 0.004 |  | - | - | - |
| WtHr | - | - | - |  | 0.214 | (0.040; 0.387) | 0.016 | - | - | - |  | 0.361 | (0.163; 0.560) | <0.001 |
| **New classification** | | | | | | | | | | | | | | |
| **Variable** | **Model A- BMI** | | |  | **Model B - WtHr** | | | **Model A- BMI** | | |  | **Model B - WtHr** | | |
|  | **b** | **(95% CI)** | **P** |  | **b** | **(95% CI)** | **P** | **b** | **(95% CI)** | **P** |  | **b** | **(95% CI)** | **P** |
| Intercept | 24.629 | (14.418; 34.839) | <0.001 |  | 29.961 | (19.688; 40.235) | <0.001 | 24.629 | (14.418; 34.839) | <0.001 |  | 18.636 | (7.103; 30.168) | 0.002 |
| MUO vs MHO | -0.292 | (-3.272; 2.687) | 0.847 |  | 0.522 | (-2.462; 3.506) | 0.731 | -0.292 | (-3.272; 2.687) | 0.847 |  | -0.181 | (-3.109; 2.746) | 0.903 |
| Puberty | 1.729 | (-1.079; 4.538) | 0.226 |  | 0.444 | (-1.890; 2.779) | 0.708 | 1.729 | (-1.079; 4.538) | 0.226 |  | 2.841 | (-0.012; 5.694) | 0.051 |
| Age (years) | -0.254 | (-0.783; 0.276) | 0.346 |  | -0.429 | (-0.888; 0.029) | 0.066 | -0.254 | (-0.783; 0.276) | 0.346 |  | -0.586 | (-1.107; -0.066) | 0.027 |
| BMI (z-score) | 6.456 | (2.599; 10.314) | 0.001 |  | - | - | - | 6.456 | (2.599; 10.314) | 0.001 |  | - | - | - |
| WtHr | - | - | - |  | 0.217 | (0.042; 0.391) | 0.015 | - | - | - |  | 0.394 | (0.198; 0.590) | <0.001 |

b indicates multivariate coefficient; CI, confidence interval; MUO, metabolically unhealthy obese; MHO, metabolically healthy obese; BMI, body mass index; WtHr, waist-to-height ratio

**Table S4**. Effect of metabolically unhealthy obese phenotype, gender, age, BMI (Model A) or waist-to-height ratio (Model B) on left ventricular hypertrophy separately in males (left panel) and females (right panel) by a multiple logistic regression model.

| **Males (N = 244)** | | | | | | | | **Females (N = 215)** | | | | | | |
| --- | --- | --- | --- | --- | --- | --- | --- | --- | --- | --- | --- | --- | --- | --- |
| **Standard classification** | | | | | | | | | | | | | | |
| **Variable** | **Model A- BMI** | | |  | **Model B - WtHr** | | | **Model A- BMI** | | |  | **Model B - WtHr** | | |
|  | **OR** | **(95% CI)** | **P** |  | **OR** | **(95% CI)** | **P** | **OR** | **(95% CI)** | **P** |  | **OR** | **(95% CI)** | **P** |
| MUO vs MHO | 0.997 | (0.555; 1.798) | 0.991 |  | 1.058 | (0.596; 1.891) | 0.848 | 2.026 | (1.116; 3.727) | 0.021 |  | 1.897 | (1.041; 3.496) | 0.038 |
| Puberty | 0.919 | (0.435; 1.926) | 0.824 |  | 1.060 | (0.497; 2.251) | 0.879 | 1.522 | (0.641; 3.661) | 0.343 |  | 1.861 | (0.763; 4.624) | 0.175 |
| Age (years) | 1.008 | (0.866; 1.174) | 0.914 |  | 0.951 | (0.818; 1.104) | 0.507 | 0.928 | (0.785; 1.093) | 0.376 |  | 0.893 | (0.756; 1.050) | 0.176 |
| BMI (z-score) | 2.646 | (0.994; 7.611) | 0.059 |  | - | - | - | 1.657 | (0.505; 5.435) | 0.401 |  | - | - | - |
| WtHr | - | - | - |  | 1.043 | (0.987; 1.104) | 0.137 | - | - | - |  | 1.067 | (1.003; 1.137) | 0.043 |
| **New classification** | | | | | | | | | | | | | | |
| **Variable** | **Model A- BMI** | | |  | **Model B - WtHr** | | | **Model A- BMI** | | |  | **Model B - WtHr** | | |
|  | **OR** | **(95% CI)** | **P** |  | **OR** | **(95% CI)** | **P** | **OR** | **(95% CI)** | **P** |  | **OR** | **(95% CI)** | **P** |
| MUO vs MHO | 0.933 | (0.353; 2.658) | 0.891 |  | 1.061 | (0.410; 2.972) | 0.905 | 0.811 | (0.333; 2.066) | 0.650 |  | 0.779 | (0.317; 1.993) | 0.590 |
| Puberty | 0.917 | (0.433; 1.922) | 0.819 |  | 1.059 | (0.497; 2.249) | 0.881 | 1.578 | (0.668; 3.777) | 0.300 |  | 2.021 | (0.832; 5.019) | 0.124 |
| Age (years) | 1.010 | (0.870; 1.174) | 0.893 |  | 0.953 | (0.821; 1.104) | 0.518 | 0.959 | (0.814; 1.127) | 0.615 |  | 0.906 | (0.768; 1.065) | 0.236 |
| BMI (z-score) | 2.690 | (0.996; 7.845) | 0.059 |  | - | - | - | 2.310 | (0.721; 7.562) | 0.160 |  | - | - | - |
| WtHr | - | - | - |  | 1.043 | (0.986; 1.104) | 0.140 | - | - | - |  | 1.083 | (1.019; 1.153) | 0.012 |

OR indicates odds ratio; CI, confidence interval; MUO, metabolically unhealthy obese; MHO, metabolically healthy obese; BMI, body mass index; WtHr, waist-to-height ratio
